# FANCJ/BACH1 Acetylation at Lysine 1249 Regulates the DNA Damage Response

**DOI:** 10.1371/journal.pgen.1002786

**Published:** 2012-07-05

**Authors:** Jenny Xie, Min Peng, Shawna Guillemette, Steven Quan, Stephanie Maniatis, Yuliang Wu, Aditya Venkatesh, Scott A. Shaffer, Robert M. Brosh, Sharon B. Cantor

**Affiliations:** 1 Department of Cancer Biology, University of Massachusetts Medical School, Worcester, Massachusetts, United States of America; 2 Proteomics and Mass Spectrometry Facility and Department of Biochemistry and Molecular Pharmacology, University of Massachusetts Medical School, Worcester, Massachusetts, United States of America; 3 Laboratory of Molecular Gerontology, National Institute on Aging, NIH Biomedical Research Center, National Institutes of Health, Baltimore, Maryland, United States of America; Baylor College of Medicine, United States of America

## Abstract

BRCA1 promotes DNA repair through interactions with multiple proteins, including CtIP and FANCJ (also known as BRIP1/BACH1). While CtIP facilitates DNA end resection when de-acetylated, the function of FANCJ in repair processing is less well defined. Here, we report that FANCJ is also acetylated. Preventing FANCJ acetylation at lysine 1249 does not interfere with the ability of cells to survive DNA interstrand crosslinks (ICLs). However, resistance is achieved with reduced reliance on recombination. Mechanistically, FANCJ acetylation facilitates DNA end processing required for repair and checkpoint signaling. This conclusion was based on the finding that FANCJ and its acetylation were required for robust RPA foci formation, RPA phosphorylation, and Rad51 foci formation in response to camptothecin (CPT). Furthermore, both preventing and mimicking FANCJ acetylation at lysine 1249 disrupts FANCJ function in checkpoint maintenance. Thus, we propose that the dynamic regulation of FANCJ acetylation is critical for robust DNA damage response, recombination-based processing, and ultimately checkpoint maintenance.

## Author Summary

The BRCA1–Fanconi anemia (FA) pathway is required for both tumor suppression and cell survival, particularly following treatment with DNA damaging agents that induce DNA interstrand crosslinks (ICLs). ICL processing by the BRCA–FA pathway includes promotion of homologous recombination (HR) and DNA damage tolerance through translesion synthesis. However, little is known about how the BRCA–FA pathway or these ICL processing mechanisms are regulated. Here, we identify acetylation as a DNA damage–dependent regulator of the BRCA–FA protein, FANCJ. FANCJ acetylation at lysine 1249 is enhanced by expression of the histone acetyltransferase CBP and reduced by expression of histone deacetylases HDAC3 or SIRT1. Furthermore, acetylation on endogenous FANCJ is induced upon treatment of cells with agents that generate DNA lesions. Consistent with this post-translation event regulating FANCJ function during cellular DNA repair, preventing FANCJ acetylation skews ICL processing. Cells have reduced reliance on HR factor Rad54 and greater reliance on translesion synthesis polymerase polη. Our data indicate that FANCJ acetylation contributes to DNA end processing that is required for HR. Furthermore, resection-dependent checkpoint maintenance relies on the dynamic regulation of FANCJ acetylation. The implication of these findings is that FANCJ acetylation contributes to DNA repair choice within the BRCA–FA pathway.

## Introduction

The hereditary breast cancer associated gene product, BRCA1 is an essential tumor suppressor. To promote genomic stability, BRCA1 interacts with multiple protein partners. In particular, through its C-terminal BRCT repeats, BRCA1 directly interacts with Abraxas, CtIP and FANCJ (also known as BRIP1 or BACH1 (BRCA1-associated C-terminal helicase 1)). These BRCT-interacting proteins contribute to the function of BRCA1 in the DNA damage response (DDR). Abraxas serves to localize BRCA1 to sites of DNA damage and CtIP promotes the initiation of DNA end resection, which is critical for HR [1]–[3]. FANCJ also participates in localizing BRCA1 to sites of DNA damage, in DNA repair, and in checkpoint signaling; however, its distinct function is less clear.

Elucidating how FANCJ functions in the DDR is important, as mutations in the *FANCJ* gene are associated with hereditary breast cancer as well as with the rare cancer prone syndrome Fanconi anemia (FA) within the FANCJ patient complementation group (FA-J) [4]. As a DEAH-family helicase, it is expected that FANCJ metabolizes DNA substrates to facilitate DNA repair. Consistent with this idea, recombinant-FANCJ is a 5′-3′ helicase and translocase that can unwind D-loops and displace RAD51 [5]. In cells, FANCJ also localizes to sites of DNA damage. Furthermore, when FANCJ is absent, catalytically inactive, or lacks BRCA1 binding, cells display defects in double strand break repair (DSBR) and HR [6]–[9]. Recently, FANCJ was identified as a factor essential for maintaining the DNA damage induced checkpoint in response to ionizing radiation [10]. Despite these findings, FANCJ-deficient cells are only mildly sensitive to agents that induce DSBs [11].

To explain these findings, it has been proposed that FANCJ functions in DSBR, but has a more significant role in processing replication forks stalled at lesions, such as DNA interstrand crosslinks (ICLs). In support of this idea, FANCJ-null cells, similar to other FA patient cells, are extremely sensitive to agents that induce ICLs, such as cisplatin, melphalan, or mitomycin C (MMC) [7], [12], [13]. This sensitivity is reversed by complementation of FA-J cells with wild-type FANCJ (FANCJ^WT^), but not with catalytically inactive FANCJ mutants [6], [8], [14]. Interestingly, the mechanism by which FANCJ mediates ICL processing is regulated by BRCA1 binding. HR is favored when BRCA1 binds FANCJ. When BRCA1 binding is prevented, lesion bypass is favored by a mechanism requiring the translesion synthesis polymerase polη [9]. Thus, complementation of FA-J cells with a BRCA1-interaction defective mutant FANCJ^S990A^ reverses ICL sensitivity but does not fully restore FANCJ function.

Here, we present evidence that FANCJ contributes to lesion processing by promoting a robust DDR. Essential for this function is FANCJ acetylation on a specific lysine residue. As such, preventing FANCJ acetylation suppresses DNA end resection that normally serves to engage recombination-based processing. Thus, both BRCT-interacting proteins, CtIP and FANCJ undergo DNA damage induced changes in acetylation that further regulates their function in the DDR to promote genomic stability.

## Results

### FANCJ is acetylated by CBP and deacetylated by HDAC3 or SIRT1

**Figure 1:**
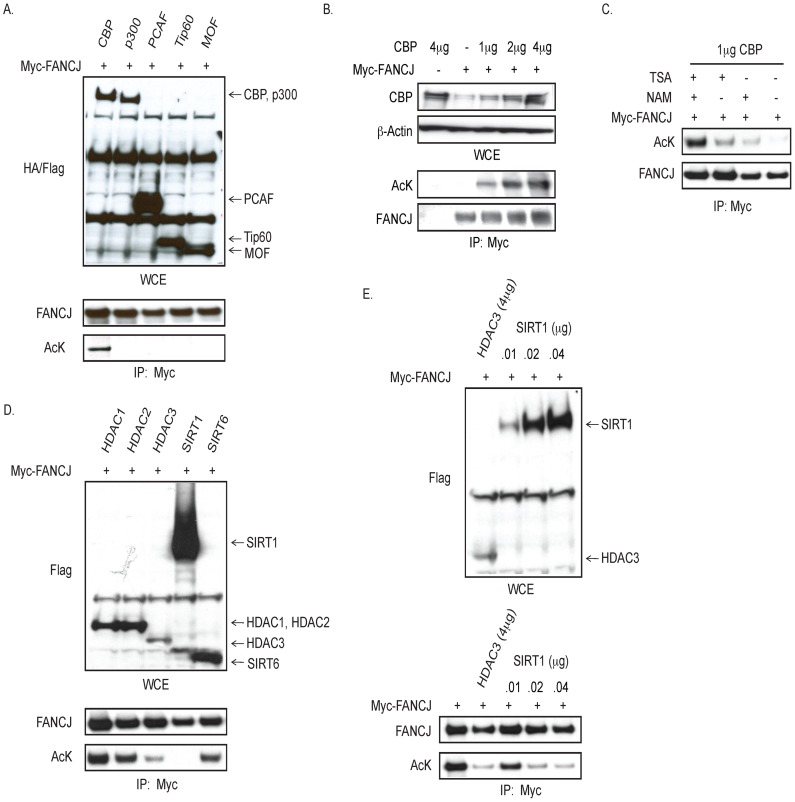
CBP promotes, and HDAC3 and SIRT1 reduce, FANCJ acetylation. A. CBP expression promotes FANCJ acetylation. Myc-tagged FANCJ was co-transfected with HA- or FLAG-tagged acetyltransferase (CBP, p300, PCAF, Tip60, or MOF) into 293T cells. As detected by immunoblot of whole cell extracts (WCE), acetyltransferases were expressed (upper panel), however only CBP promoted FANCJ acetylation as shown by FANCJ immunoprecipitation (lower panel). B. FANCJ acetylation is induced by CBP in a dose dependent manner. Myc-tagged FANCJ was co-transfected with increasing amounts of CBP and lysates were used for immunoblot with the indicated antibodies. C. FANCJ deacetylation was prevented by both TSA and NAM. 293T cells co-expressing Myc-tagged FANCJ and CBP were exposed to TSA, NAM, TSA+NAM, or neither and lysates were used for immunoblot with the indicated antibodies. D. FANCJ was deacetylated by both HDAC3 and SIRT1. Myc-tagged FANCJ was co-transfected with CBP and Flag-tagged deacetylase (HDAC1, HDAC2, HDAC3, SIRT1, or SIRT6) into 293T cells and lysates were immunobloted with the indicated antibodies. E. When SIRT1 (0.01 µg) and HDAC3 (4 µg) were similarly expressed (upper panel), HDAC3 promoted more FANCJ deacetylation (lower panel). Cell lysates were collected and analyzed for expression and/or acetyation following immunoprecipitation with the indicated antibodies.

As observed for CtIP, FANCJ binds directly to the BRCT domains of BRCA1 [6], [9], [15]. Given that CtIP function is inactivated by acetylation [16], we addressed whether FANCJ was similarly modified. For this analysis, myc-tagged FANCJ was co-transfected with various Flag- or HA-tagged histone acetyltransferases. In an immunoblot probed with a pan-acetyl lysine antibody, we found that the precipitated FANCJ was acetylated only when CBP was over-expressed (Figure 1A). Moreover, FANCJ acetylation was induced by CBP in a dose dependent manner (Figure 1B).

FANCJ acetylation was preserved most effectively by the inclusion of two types of deacetylase inhibitors, trichostatin-A (TSA) and nicotinamide (NAM) (Figure 1C). Thus, we considered that more than one class of histone deacetylase (HDAC) could deacetylate FANCJ. TSA inactivates class 1 and class II HDACs, whereas NAM inactivates the nicotinamide adenoine dinucleotide (NAD^+^)-dependent sirtuin (class III) family of HDACs (including SIRT1 to SIRT7) [17]. FANCJ acetylation was reduced more when either Flag-tagged-HDAC3 or SIRT1 were overexpressed in 293T cells than observed upon overexpression of HDAC1, HDAC2, or SIRT6 (Figure 1D). Titration of the SIRT1 expression vector transfected into 293T cells revealed that 0.01 µg of the SIRT1 construct matched the expression level of 4 µg of the HDAC3 construct. At this similar level of expression, HDAC3 more efficiently deactylated FANCJ than did SIRT1 (Figure 1E). Together, these data implicate that FANCJ can be acetylated by CBP and deacetyated by HDAC3 as well a SIRT1 when over-expressed.

### FANCJ is acetylated at lysine residue 1249

**Figure 2:**
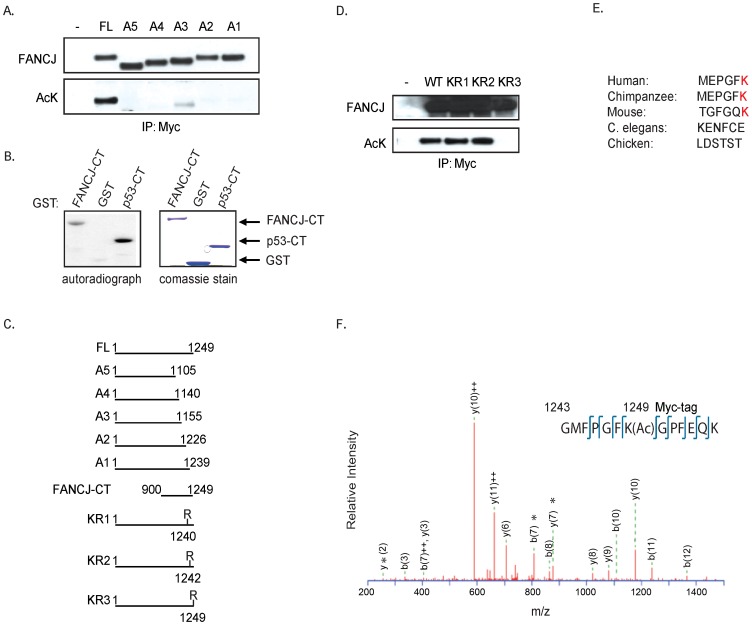
FANCJ is acetylated at lysine 1249. A. The C-terminus of FANCJ is required for acetylation. Myc-tagged FANCJ mutant constructs were co-expressed with CBP into 293T cells. Cell lysates were collected and analyzed for expression and/or acetylation following immunoprecipitation with the indicated antibodies. B. The FANCJ C-terminus is acetylated *in vitro*. The recombinant histone acetyltransferase (HAT) domain of p300 was incubated with recombinant FANCJ C-terminal (CT) or p53-CT in the presence of 3H-acetyl CoA. Reaction products were separated by SDS-PAGE and analyzed by autoradiography. Expression of recombinant proteins was determined by Coomassie staining. C. Schematic presentation of wild-type and truncation mutations of FANCJ. D. Lysine 1249 is required for FANCJ acetylation. The Myc-tagged FANCJ mutant constructs noted were co-expressed with CBP into 293T cells and cell lysates were collected and analyzed for expression and/or acetylation following immunoprecipitation with the indicated antibodies. E. Sequence alignment of last 6 residues found in distinct FANCJ species. F. Confirmation of the K1249 acetylation is shown by tandem mass spectrum of FANCJ peptide (amino acid 1243–1249 with Myc tag). *Ions validating localization of acetylation site.

To identify the FANCJ acetylation site(s), myc-tagged C-terminal FANCJ truncation mutants were co-transfected with CBP into 293T cells. By Immunoblot analysis using the pan-acetyl antibody, we found that acetylation of FANCJ required amino acids 1239 to 1249 (Figure 2A, 2C). Consistent with this region being modified, a C-terminal domain of FANCJ similar to a C-terminal p53 control was acetylated *in vitro* by a HAT-domain protein (Figure 2B, 2C). To determine, which of three lysine (K) residues in this C-terminal region were required for acetylation, we generated three independent FANCJ mutant constructs that converted lysines 1240, 1242, or 1249 to arginine (R). Further transfection experiments revealed that the K1249 was the dominant site for FANCJ acetylation, a lysine that is not conserved in chicken or *C. elegans* FANCJ species (Figure 2D, 2E).

Next, we sought to provide more conclusive evidence that CBP-induced acetylation on FANCJ was at the K1249 site. We purified FANCJ from 293T cells transfected with a C-terminal myc-tagged FANCJ^WT^ or the FANCJ^K1249R^ mutant species by immunoprecipitation using a myc antibody. Isolated proteins were then digested with trypsin and subjected to tandem mass spectrometry analysis (LC-MS/MS). FANCJ-derived peptides covering the entire sequence were analyzed, and acetylation sites were identified using MASCOT search algorithm. Most of the acetylated lysine residues were detected in overlapping peptides derived from at least two independent protein preparations. In the FANCJ^WT^, one of these sites was K1249 (Figure 2F). Interestingly, even though by antibody detection, the FANCJ^K1249R^ mutant scores unmodified as in Figure 2D; FANCJ^WT^ and FANCJ^K1249R^ mutant had three additional acetylation marks detected by mass spectrometry (Figure S1). Furthermore, the K1249R mutant had five additional acetylated lysines not found in wild-type FANCJ, suggesting that these sites are not available when K1249 is acetylated (Figure S1). Thus, immunoblot and mass spectrometry analysis confirm that the very last amino acid of FANCJ, lysine 1249 is acetylated.

### FANCJ acetylation is enhanced by certain forms of DNA damage

**Figure 3:**
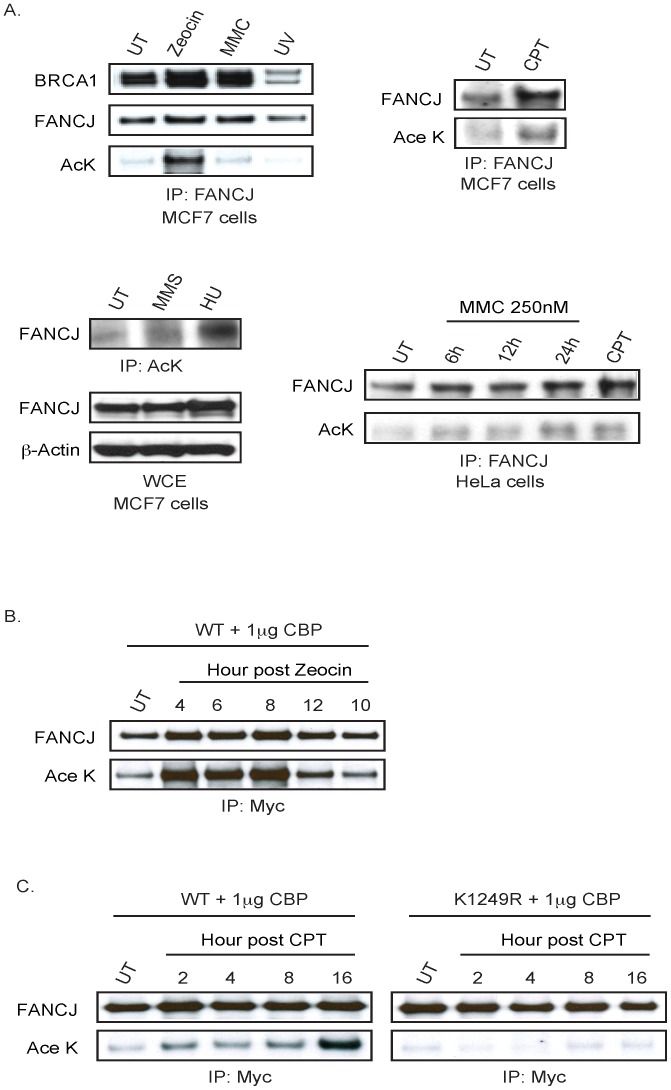
FANCJ acetylation is induced after DNA damage. A. Endogenous FANCJ is acetylated in response DNA damage. MCF7 and HeLa cells were left untreated (UT) or treated with zeocin (6.25 µg/ml for 1 h), MMC (250 nM for 1 h), UV (30 J/m^2^), MMS (300 µg/ml for 4 h), HU (1 mM for 24 h), or CPT (1 µM for 1 h). Cell lysates were collected at distinct times post damage (zeocin 24 h, MMC 24 h or as indicated, UV 6 h, CPT 24 h, MMS 4 h, and HU 24 h) and analyzed for expression and/or acetylation following immunoprecipitation with the indicated antibodies. B. Exogenous FANCJ is acetylated on lysine 1249 in response to DNA damage. Myc-tagged FANCJ wild-type or mutant species and CBP were co-transfected into 293T cells and left untreated (UT) or treated with zeocin (12.5 µg/ml for 1 h or C. CPT (1 µM for 1 h). Cells were processed at different time points post DNA damage and analyzed for expression and/or acetylation following immunoprecipitation with the indicated antibodies.

Given that DNA damage reduces CtIP acetylation [16], we addressed whether DNA damage could alter FANCJ acetylation. Endogenous FANCJ acetylation was enhanced in MCF7 cells treated with zeocin, camptothecin (CPT), or hydroxyurea (HU) as compared to ultraviolet radiation (UV), MMC, or methyl methanesulfonate (MMS) at the dose and time-post treatment analyzed (Figure 3A). Notably, zeocin had a more robust induction of FANCJ acetylation despite the dose of zeocin, CPT, or UV having similar affect on cell survival (Figure S2; data not shown). As found previously, DNA damage did not measurably alter FANCJ co-precipitation with BRCA1 with the exception of UV damage, which could reflect the UV-induced BRCA1 degradation [11], [18] (Figure 3A). DNA damage also induced FANCJ acetylation in HeLa cells, in response to not only CPT, but also MMC (Figure 3A). In response to DNA damage, we also noted that FANCJ protein levels were sometimes enhanced (Figure 3A). To clarify whether acetylation or our ability to detect acetylation was induced by DNA damage, we sought to induce DNA damage in cells in which our ability to detect FANCJ acetylation was not limiting. Indeed, the amount of acetylation on similar levels of exogenous FANCJ^WT^ achieved with low dose CBP expression was considerably enhanced following treatment with zeocin or CPT (Figure 3B, 3C). Interactions with BRCA1 and MLH1 were not required for the CBP-induced acetylation of FANCJ, because BRCA1- and MLH1-interaction-defective mutants, FANCJ^S990A^ and FANCJ^K141/142A^ were readily modified (data not shown). In contrast, following treatment with CPT, acetylation was not detected on the FANCJ^K1249R^ mutant (Figure 3C), indicating that DNA damage-induced FANCJ acetylation requires the C-terminal K1249 residue. It remains to be determined, however if FANCJ acetylation is induced by a distinct type of DNA damage.

### FANCJ acetylation mutants are functional

**Figure 4:**
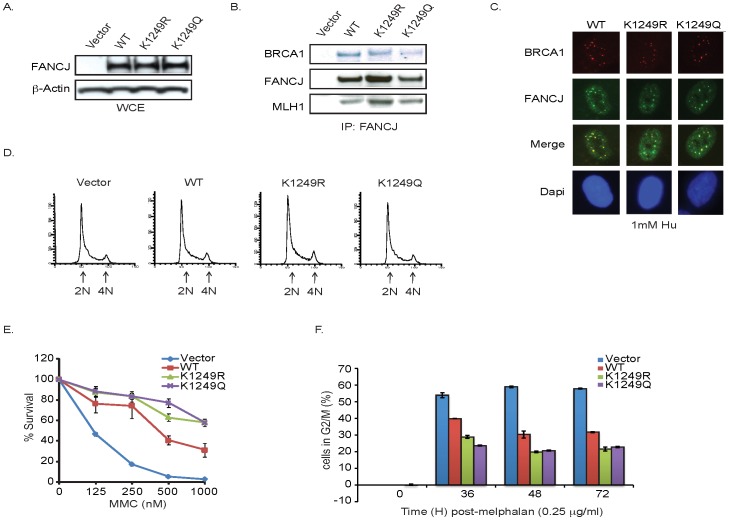
FANCJ acetylation mutants are functional. A. The acetylation mutants are expressed in FA-J cells. FA-J cells were complemented with vector, FANCJ^WT^, FANCJ^K1249R^, or FANCJ^K1249Q^. The FA-J cell lines were collected and analyzed or B. lysates were immunoprecipitated with FANCJ antibodies and immunoblot was performed with the indicated antibodies. C. The acetylation mutants localize in nuclear foci of FA-J cells. The FA-J cell lines were seeded onto 6-well plates and incubated overnight. The cells were treated with 1 mM HU and 24 h later immunoflourescence was performed with the indicated antibodies. D. The FA-J cell lines have similar cell cycle profiles. The FA-J cells lines were collected and analyzed by FACS to determine the percentage of cells with 2N and 4N DNA content. E. Expression of acetylation mutants restores MMC resistance. The FA-J cell lines were seed onto 6 well plates and incubated overnight. The cells were either left untreated or treated with increasing doses of MMC. Cells were counted 8 days later and percent survival was calculated. Data represent mean percent ± s.d. of survival from three independent experiments. F. Expression of acetylation mutants restores G2/M checkpoint exit. The FA-J cell lines were untreated or treated with 0.25 µg/ml melphalan, collected at the indicated times, and analyzed by FACS to determine the percentage of cells in G2/M. Data represent mean percent ± s.d. of survival from three independent experiments.

The enhanced FANCJ acetylation following DNA damage led us to hypothesize that this modification facilitated FANCJ function in DNA repair. To address this possibility, we made use of this lysine to arginine FANCJ^K1249R^ mutant that prevents acetylation and also generated a lysine to glutamine FANCJ^K1249Q^ mutant to structurally mimic acetylation. Consistent with these mutants being functional, the purified recombinant proteins displayed similar catalytic activities as FANCJ^WT^ (Figure S3). In addition, they were expressed at similar levels as FANCJ^WT^ in FANCJ-null FA-J cells (Figure 4A). Similar to FANCJ^WT^, FANCJ^K1249R^ and FANCJ^K1249Q^ precipitated with known FANCJ interacting partners, BRCA1 and MLH1 [6], [8] (Figure 4B). In addition, the mutants co-localized with BRCA1 in response to DNA damage and the FA-J cells expressing FANCJ^WT^ or mutants had similar asynchronous cell cycle profiles (Figure 4C, 4D). The acetylation mutants also restored MMC resistance and the ability of FA-J cells to exit from an abnormal G2/M accumulation, albeit in a manner slightly more robust than FANCJ^WT^ (Figure 4E, 4F). Together, these findings suggested that the mutants were enzyme active and functional *in vivo*; however the mechanism by which the FANCJ mutants restore ICL resistance could be distinct from FANCJ^WT^.

### FANCJ acetylation contributes to the mechanism of lesion processing

**Figure 5:**
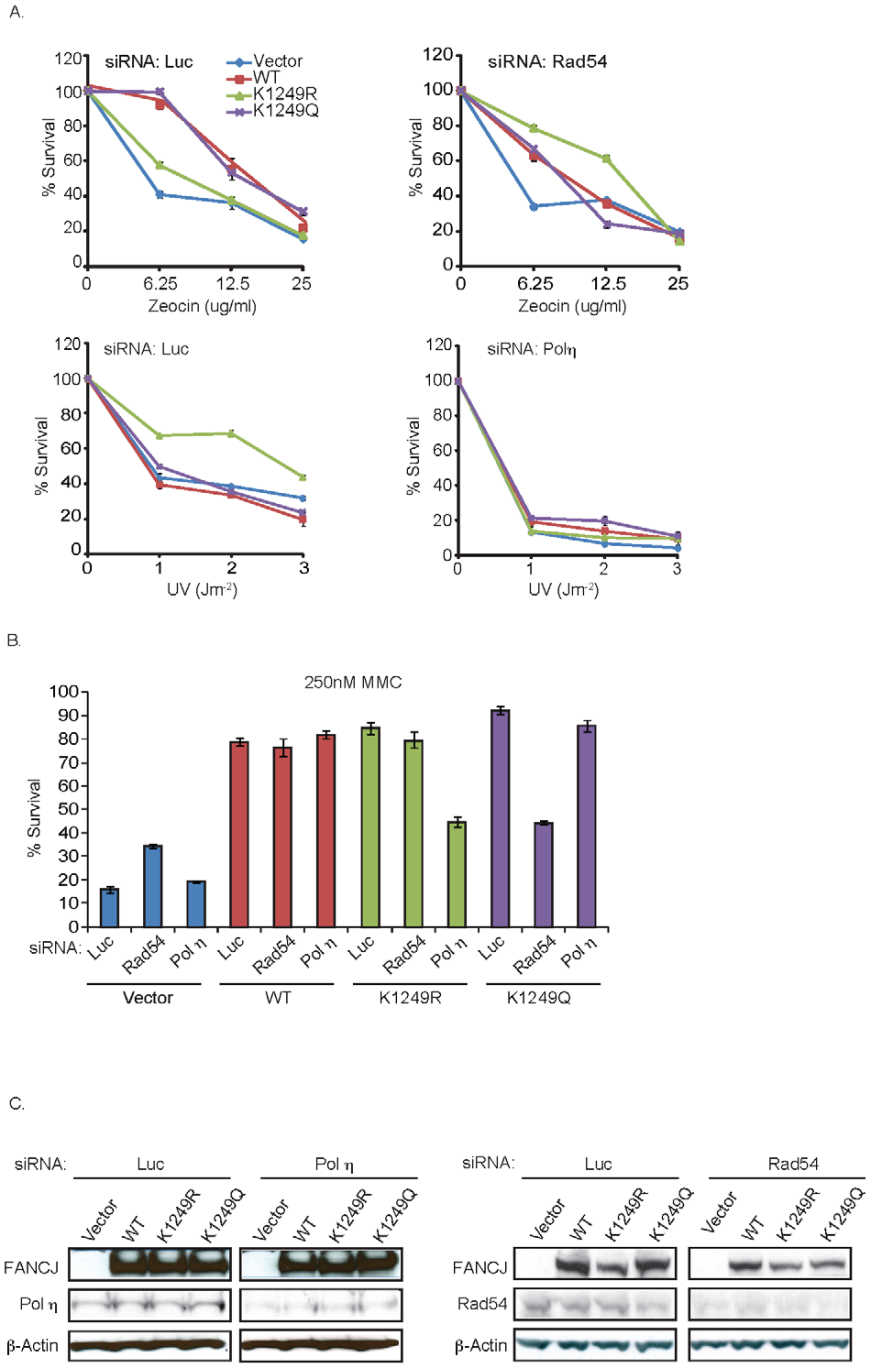
FANCJ^K1249R^ or FANCJ^K1249Q^ promotes polη- or Rad54-dependent repair, respectively. A. FA-J cells expressing acetylation mutants have a distinct response and reliance on repair or tolerance factors for DNA damage survival. The FA-J cell lines were transfected with siRNA against Luc, Polη, or Rad54. The cells were treated with indicated doses of zeocin, UV, or as in B., with MMC and the percent survival was calculated 8 days later. Data represent mean percent ± s.d. of survival from three independent experiments. C. Cells were collected and analyzed for expression with the indicated antibodies.

Previously, complementation of FA-J cells with a BRCA1-binding defective mutant, FANCJ^S990A^ gave the semblance of FANCJ^WT^ function. In particular, MMC resistance was restored [8]. However, in contrast to FANCJ^WT^, FANCJ^S990A^ provides resistance to MMC by a mechanism dependent on the DNA damage tolerance pathway. Within this tolerance pathway, translesion synthesis polymerases can bypass DNA lesions such as unhooked ICLs and intra-strand crosslinks generated by UV, but not DSBs generated by zeocin. Evidence that FANCJ^S990A^ skewed lesion processing towards DNA damage tolerance was based on several findings. First, the sensitivity to MMC in these cells was restored upon depletion of the essential tolerance factor, Rad18 or the translesion polymerase polη, but not upon depletion of the HR protein, Rad54. Second, in comparison to FANCJ^WT^, cells expressing FANCJ^S990A^ were hyper-resistant to UV, a phenotype that was reversed upon polη-depletion. Third, in comparison to FANCJ^WT^, FANCJ^S990A^-expressing cells were sensitive to zeocin, indicating reduced DSBR [9]. Thus, we sought to determine whether similar to the BRCA1-binding mutant, the acetylation mutants also functioned differently from FANCJ^WT^. To test this idea, the FA-J cell lines were left untreated or treated with increasing doses of MMC, zeocin, or UV. In comparison to the other FA-J cell lines, the FA-J cell line expressing the acetylation mutant FANCJ^K1249R^ was hyper-resistant to UV, but unable to restore normal levels of zeocin resistance. In contrast, the FA-J cell line expressing the acetylation mimic FANCJ^K1249Q^ displayed greater resistance to zeocin (Figure 5A; Figure S2). Thus, in response to UV and zeocin, cells expressing the acetylation mutants are distinct from each other as well as from cells expressing FANCJ^WT^.

To further validate these results, we targeted recombination or DNA damage tolerance pathways by using siRNA reagents to Rad54 or polη. Significantly, depletion of Rad54 suppressed the zeocin resistance of the FA-J cell line expressing FANCJ^K1249Q^ (Figure 5A, 5C). Likewise, depletion of polη suppressed the UV hyper-resistance of the FA-J cell line expressing FANCJ^K1249R^ (Figure 5A, 5C). Furthermore, depletion of polη, but not Rad54 reversed the MMC resistance of the FA-J cell line expressing FANCJ^K1249R^ (Figure 5B). In contrast, depletion of Rad54, but not polη reduced the MMC resistance of the FA-J cell line expressing FANCJ^K1249Q^ (Figure 5B). Together, these results indicate that the acetylation of FANCJ at lysine 1249 contributes to the mechanism of lesion processing; preventing acetylation favors DNA damage tolerance and constitutive acetylation favors recombination.

### FANCJ acetylation is required for a robust DDR

**Figure 6:**
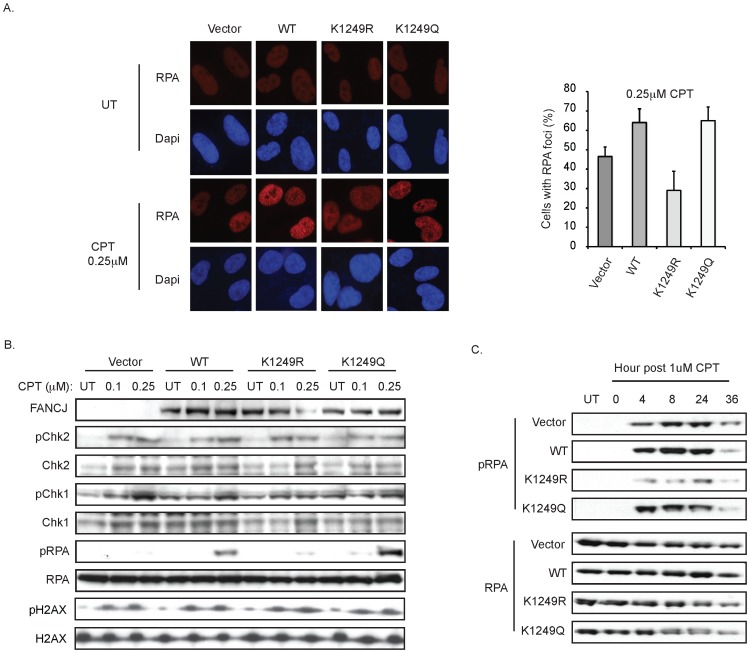
FANCJ and its acetylation at 1249 promote an RPA response. A. Deficiency in FANCJ or its acetylation impairs the CPT-induced RPA focus formation. The FA-J cell lines were seeded onto 6-well plates, incubated overnight, left untreated or treated with CPT 1 h and immunoflourescence was performed with the indicated antibodies. The percent of cells with RPA foci was quantified and graphed. Data represent mean ± s.d. from three independent experiments. B. FANCJ and its acetylation promote RPA phosphorylation at 1 h post-CPT. The complemented FA-J cell lines were either left untreated or treated for 1 h with the indicated dose of CPT and analyzed 1 h post-treatment. Cell lysates were collected, lysed, and analyzed with the indicated antibodies. C. FANCJ acetylation promotes RPA phosphorylation at times greater than 1 h post-CPT. Same as above but collected at the time points indicated.

How could FANCJ acetylation affect lesion processing? Because both CtIP and FANCJ are acetylated and directly bind to the BRCA1-BRCT domain, we speculated that FANCJ might similarly have a role in DNA end resection. In particular, the affect of CtIP acetylation on DNA end resection was analyzed in response to CPT [16]. We found RPA foci formation at 1 h post-CPT was more robust (64% and 65%) in the FANCJ^WT^ and FANCJ^K1249Q^ FA-J cell lines as compared to vector and FANCJ^K1249R^ FA-J cell lines that had 47% and 29%, respectively (Figure 6A). Thus, as measured by RPA foci formation, FANCJ^WT^ and the acetylation mimic FANCJ^K1249Q^ were more active in DNA end resection.

RPA loading onto ssDNA also leads to its subsequent phosphorylation on Ser4 and Ser8 [3]. We found that the FA-J cell lines had a similar phosphorylation of Chk2 and γ-H2AX following exposure to two different dose of CPT, indicating that FANCJ or its ability to be acetylated is not required for DSB formation in response to CPT (Figure 6B). Likewise, at 1 h post-CPT treatment, Chk1 phosphorylation was detected (Figure 6B). In contrast, RPA phosphorylation was most robust in the CPT-treated FANCJ^WT^ and FANCJ^K1249Q^ FA-J cell lines (Figure 6B). In support of these findings, reduced RPA phosphorylation was also detected in CPT-treated FANCJ-deficient U2OS cells generated by siRNA reagents (Figure S4). Furthermore, at 4–24 h post CPT treatment, we noted diminished RPA phosphorylation in FANCJ^K1249R^ as compared with FANCJ^WT^ and FANCJ^K1249Q^ FA-J cell lines (Figure 6C). At this time, RPA phosphorylation in the FANCJ^K1249R^ FA-J cells was also reduced compared to vector FA-J cells that had gained considerable RPA phosphorylation as compared to 1 h post-CPT (Figure 6B, 6C). In the response to zeocin, which induces DSBs independent of replication, RPA phosphorylation was similar in FA-J cell lines with or without FANCJ^WT^ (Figure S5). Together, these results suggest a role for FANCJ and its acetylation in DNA end resection at stalled replication forks as induced by CPT.

**Figure 7:**
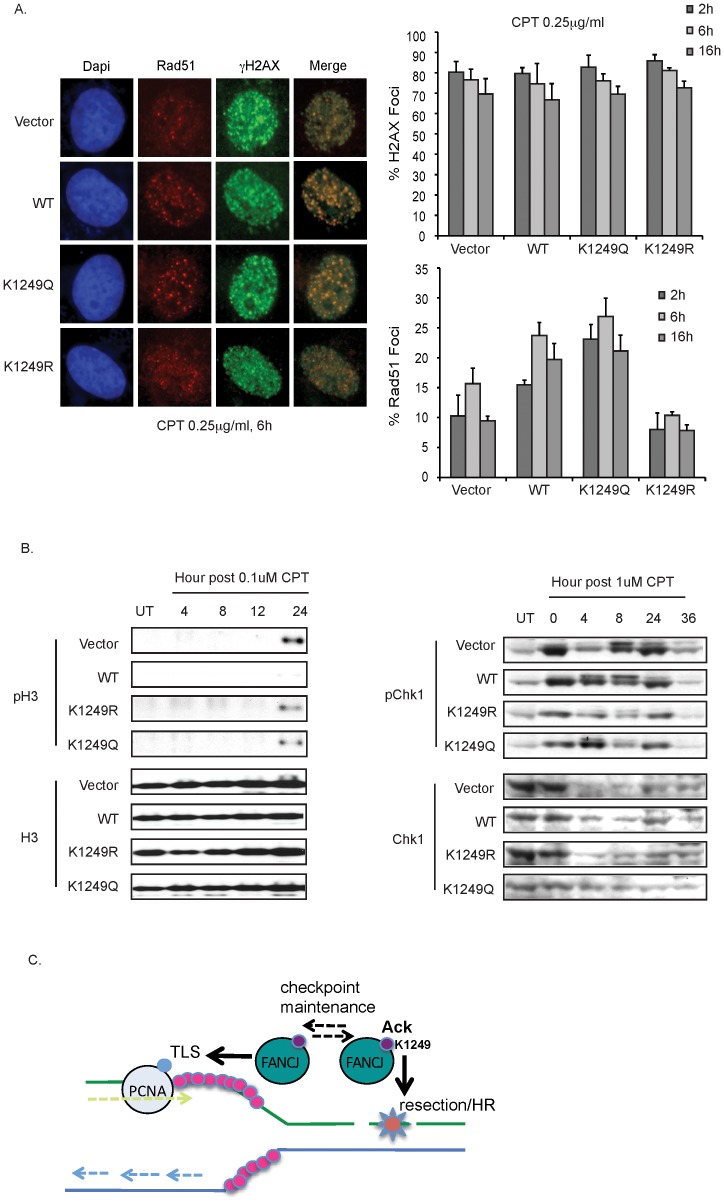
FANCJ and its dynamic regulation by acetylation promote resection-associated events. A. Deficiency in FANCJ or its acetylation impairs the CPT-induced Rad51 focus formation. The FA-J cell lines were seeded onto 6-well plates, incubated overnight, left untreated or treated with CPT 6 h and immunoflourescence was performed with the indicated antibodies. The percent of cells with γ-H2AX and Rad51 foci was quantified and graphed. Data represent mean percent ± s.d. from three independent experiments. B. Deficiency in FANCJ or its ability to be regulated by acetylation impairs the CPT-induced checkpoint response. FA-J cell lines were either left untreated or treated for 1 h with the 0.1 µM or 1 µM of CPT and analyzed at the indicated time points. Cell lysates were collected, lysed, and analyzed with the indicated antibodies. C. Model depicts function of FANCJ acetylation in the DDR. FANCJ acetylation at lysine 1249 promotes resection and HR whereas de-acetylation promotes translesion synthesis (TLS). Maintenance of the checkpoint response; however requires the dynamic regulation of FANCJ acetylation.

To address whether the contribution of FANCJ acetylation to DNA end resection was sufficient to enhance HR, we next analyzed Rad51 foci formation. In response to CPT, we found that Rad51 foci were the most robust in FA-J cells complemented with FANCJ^WT^ or the FANCJ^K1249Q^ mutant. Instead, Rad51 foci in the FA-J cells with vector or FANCJ^K1249R^ were more anemic (Figure 7A). Furthermore, a greater number of FANCJ^K1249Q^ expressing FA-J cells were positive for Rad51 foci as quantitated between 2–16 h after CPT treatment (Figure 7A). In contrast, the γ-H2AX foci did not have a significant difference between the FA-J cells lines. Thus, a greater proportion of γ-H2AX co-staining Rad51 foci were detected in FANCJ^K1249Q^ or FANCJ^WT^, as compared to vector or FANCJ^K1249R^ expressing FA-J cells (Figure 7A merge). Together, these findings demonstrate that in response to CPT, FANCJ and its acetylation at 1249 promote DNA end processing events that enhance RPA phosphorylation, and both RPA and Rad51 focal accumulation.

Given these findings and the recent identification that FANCJ promotes checkpoint maintenance [10], we considered that FANCJ acetylation could be essential for maintaining the checkpoint. Defects in checkpoint maintenance were evaluated by determining if CPT treated FA-J cells traversed prematurely to mitosis. FA-J cells lacking FANCJ^WT^ entered mitosis by 24 h post-CPT as indicated by a positive histone H3 phosphorylation (Figure 7B). These results are consistent with FANCJ acetylation supporting checkpoint maintenance. However, FA-J cells expressing FANCJ^K1249R^ or FANCJ^K1249Q^ also failed to maintain the checkpoint, showing H3 phosphorylation by 24 h (Figure 7B). Substantiating this finding, at time points greater than 4 h post-CPT treatment, both mutants had reduced Chk1 phosphorylation as compared to FA-J cells expressing FANCJ^WT^ (Figure 7B). Collectively, these findings suggest that FANCJ acetylation enhances the initial DDR to facilitate recombination-based repair and limit translesion synthesis. Checkpoint maintenance however, requires FANCJ and its dynamic regulation by acetylation (Figure 7C).

## Discussion

Here we identify acetylation as a DNA damage-dependent regulator of the BRCA-FA protein, FANCJ. We show that acetylation at lysine 1249 is a critical regulator of FANCJ function during cellular DNA repair. We analyzed the expression of two FANCJ mutants that mimicked either the constitutive deacetylated FANCJ^K1249R^ or acetylated FANCJ^K1249Q^ protein isoforms. While the mutants functioned similar to FANCJ^WT^ in several assays and restored MMC resistance and exit from an abnormal G2/M checkpoint response to FA-J cells, the mutants were distinct from FANCJ^WT^ with respect to lesion processing. Notably, FA-J cells expressing the acetylation mutants differentially relied on repair and tolerance factors for resistance to DNA damaging agents. Our findings further demonstrate that FANCJ has the ability to potentiate HR and DNA damage induced acetylation is important for this function.

Another BRCA1-BRCT interacting protein, CtIP is acetylated and functions in DNA end resection. Thus, we considered that recombination-based lesion processing by the FANCJ acetylation mimic, FANCJ^K1249Q^ resulted from a function for FANCJ acetylation in DNA end resection. To test this idea, the FA-J cells were treated with CPT, which generates breaks in S-phase. Indeed, FA-J cells expressing the acetylation mutants were distinct in the initial response to CPT. Specifically, FA-J cells expressing the FANCJ^K1249Q^, but not FANCJ^K1249R^, promoted DNA end resection post-CPT exposure as measured by presentation of RPA foci or its phosphorylation at serine residues 4 and 8. Furthermore, FA-J cells expressing the FANCJ^K1249Q^, as compared to FANCJ^K1249R^ had 2.5-fold more cells with CPT-induced Rad51 foci. This more robust DDR could reflect a role for FANCJ acetylation in loading RPA, as shown for FANCJ in response to HU [19]. Our data do not implicate a global role for FANCJ in DNA end resection given that FANCJ-deficiency did not affect the amount of RPA phosphorylation following zeocin, an agent that induces DSBs independent of replication. However, FANCJ is acetylated when cells are exposed to CPT or zeocin. Thus, DSB-induced FANCJ acetylation that is not associated with stalled or broken replication forks may contribute to some other aspect of the DDR, such as checkpoint maintenance.

In fact, we find that FANCJ as well as its acetylation are essential for checkpoint maintenance. Specifically, in the absence of FANCJ or its DNA damage induced acetylation, Chk1 phosphorylation was induced, but not maintained and correspondingly cells underwent a more rapid transit into mitosis post-CPT. Interestingly, we found that similar to FA-J cells expressing the acetylation mutant FANCJ^K1249R^, FA-J cells expressing the acetylation mimic FANCJ^K1249Q^ failed to maintain the checkpoint despite an initial DDR to CPT. Thus, some other aspect of checkpoint signaling is perturbed in FA-J cells that express the acetylation mimic. Perhaps this mutant fails to mediate a protein interaction or act upon a DNA substrate important for checkpoint maintenance. Instead FANCJ acetylation could serve as a switch, in which acetylation and de-acetylation is essential to maintain the checkpoint (Figure 7C). Consistently, a role for FANCJ in checkpoint maintenance was reported in a recent study [10].

It follows that defects in initiating the DDR, engaging HR, and maintaining the checkpoint impact cellular DNA damage resistance. Reduced DNA repair and/or checkpoint maintenance defects could explain why FA-J cells expressing the acetylation mutant FANCJ^K1249R^ were sensitive to zeocin. Defects in repair and in maintaining the checkpoint may not increase cellular sensitivity if backup lesion processing mechanisms serve to process or bypass the lesion. Compensatory pathways could explain the lack of CPT-sensitivity in the FA-J cells with or without acetylation mutants (Figure S2). In support of this idea, our data reveal that FA-J cells expressing the acetylation mutant were resistant to DNA damage by relying on tolerance factors. As such, depletion of polη in FA-J cells expressing the non-acetylatable FANCJ^K1249R^ mutant reversed the UV and MMC resistance. Instead, FA-J cells expressing the acetylation mimic FANCJ^K1249Q^ maintained zeocin and MMC resistance in a Rad54-dependent manner. These findings suggest that the toxicity to ICLs lesions as found in cells deficient for FANCJ is avoided because FANCJ enzyme active acetylation mutants facilitate recombination in S phase or translesion synthesis bypass of unhooked ICL lesions perhaps in mitosis. In the absence of a maintained checkpoint, however recombination similar to translesion synthesis bypass is likely to be error-prone.

Previously, we found that BRCA1 binding to FANCJ altered FANCJ function in HR and translesion synthesis pathways. Indeed, we find that similar to FA-J cells expressing the acetylation FANCJ^K1249R^ mutant, FA-J cells expressing the BRCA-interaction defective mutant, FANCJ^S990A^ were hyper-resistant to UV induced damage, sensitive to zeocin induced damage, and relied on polη for MMC resistance [7]. Data also indicate that similar to FANCJ^K1249R^, the FANCJ^S990A^ mutant fails to maintain the checkpoint. In response to melphalan treatment, FA-J cells expressing the FANCJ^S990A^ mutant, as compared to FANCJ^WT^, underwent a reduced and more rapid G2/M checkpoint exit [7]. These similar outcomes do not reflect common defects in BRCA1 binding or acetylation. Indeed, the FANCJ^S990A^ mutant was acetylated upon co-transfection of CBP to levels similar to those observed for FANCJ^WT^ (data not shown). Moreover, co-precipitation experiments demonstrated that the FANCJ^K1249R^ mutant bound BRCA1 as well as FANCJ^WT^. Thus, BRCA1 binding and acetylation of FANCJ may be distinct events. Nevertheless, defects in BRCA1 binding at serine 990 or acetylation at lysine 1249 could have similar outcomes for FANCJ function because both mutants fail to maintain a robust checkpoint and Rad51-based repair is reduced [9].

A stalled replication fork with exposed single stranded and double stranded regions could provide an ideal DNA substrate for FANCJ. Indeed, FANCJ requires several nucleotides for binding and metabolizing DNA [20]. FANCJ function in replication fork processing could also be similar to other 5′-3′ DNA helicase/translocases such as *Ecoli* RecD and yeast Rad3. Rad3 facilitates exonucleolytic degradation of DNA ends, which restricts recombination between short homologous sequences [21]. Interestingly, RecD regulates resection and recombination by changes in helicase speed, which can also facilitate a polymerase swap, in which bypass polymerases diminish fork break down [22]. Conceivably, enhanced FANCJ enzyme activity or altered substrate preference due to acetylation could generate more single-stranded DNA to elicit checkpoint responses such as RPA loading as proposed [19]. Alternatively, checkpoint maintenance could require reduced FANCJ enzyme activity so that FANCJ does not displace proteins from lesions, such as RAD51 or interacting partners BRCA1, RPA and BLM helicase [6], [23]–[25]. In this context, it is worth noting that changes in motor speed have been associated with FANCJ clinical mutants. The breast cancer associated mutant, M299I is enzyme activating and both unwinds and translocates DNA more efficiently than FANCJ^WT^, whereas the P47A mutant is enzyme inactivating [26], [27]. Whether changes in FANCJ function derive from acetylation and/or partners that bind via this modification remains to be determined. Furthermore, based on our current data, it is unclear if distinct DNA lesions selectively induce FANCJ acetylation.

In summary, our findings indicate that FANCJ has the ability to potentiate HR through dual roles in DNA end processing and checkpoint maintenance. These two functions require FANCJ lysine 1249, a site not conserved in FANCJ orthologues such as chicken FANCJ and *C. elegans* Dog-1. Interestingly, unlike in human cells, FANCJ does not function in HR in chicken and C-elegans systems [28], [29]. It is not surprising that regulators of FANCJ acetylation state, HDACIII, SIRT1, and CBP have roles in DNA repair and genomic stability [30]–[32]. It remains to be determined, however, whether associated repair defects are related to failure to regulate FANCJ acetylation. Complicating this analysis, HDACIII, SIRT1, and CBP have many other histone and non-histone protein substrates that also have role in DNA repair and genomic stability. For example, SIRT1 deacetylation plays an important role in regulating the function of DNA double strand break repair proteins, such as Ku70 [33], WRN [34], and NBS1 [35]. Moreover, p300/CBP functions to regulate the activities of multiple proteins at the replication fork including PCNA [36]. CBP also regulates the activity of other helicases, including WRN [37]. Whether HDAC or HAT associated defects derive from a failure to regulate FANCJ acetylation will be an important question for future studies.

## Materials and Methods

### Cell lines

MCF7, HeLa, and 293T cells were grown in DMEM supplemented with 10% fetal bovine serum and penicillin/streptomycin (100 U/mL each). FA-J (EUFA30-F) cells were cultured with 15% fetal bovine serum and penicillin/streptomycin (100 U/mL each). FA-J cells were infected with the POZ retroviral vector [38] containing no insert, WT, K1249R, or K1249Q FANCJ inserts. Stable FA-J POZ cell lines were selected as before [8].

### Immunoprecipitation and Western blot assays

Cells were harvested, lysed, and processed for Western blot analysis as described previously using an NETN lysis buffer (20 mM Tris, 150 mM NaCl, 1 mM EDTA, and 0.5% NP-40) containing 10 mM NaF and 1 mM NaVO_3_ [7]. For acetylation detection, unless otherwise noted cells were lysed with 150 mM NETN buffer supplemented with 10 µM TSA and 5 mM nicotinamide. For γ-H2AX detection, cell pellets were collected and dissolved and boiled in 2× lysis buffer (50 mM Tris pH 6.8, 2% SDS, 1% B-ME). Antibodies used for immunoprecipitation (IP) and Western blot assays include FANCJ polyclonal Abs E67 [26], β-Actin (Sigma), pRPA S3/4 (Bethyl), RPA (Bethyl), pChk1 S317 (Bethyl), Chk1 (Bethyl), pChk2 (Cell signaling), Chk2 (Cell Signaling), γ-H2AX S139 (Millipore), H2AX (Bethyl), Flag (Sigma), HA (12C4), pan-acetylated lysine (Cell signaling), MLH1 (BD Bioscience), BRCA1 monoclonal (ms110), pH 3 (Millipore), H3 (Abcam), polη (Abcam), Rad54 (Abcam), Rad51 (Abcam), and Myc monoclonal (9E10).

### Cell cycle progression assay

FA-J stable cell lines were either mock treated or treated with 0.25 µg/ml of melphalan (Sigma) and incubated for various times. Cells were fixed with 90% methanol in PBS overnight and then incubated 10 min with PBS containing 30 µg/ml DNase-free RNase A and 50 µg/ml propidium iodide. 1×10^4^ cells were analyzed using a FACs Calibur instrument (Becton-Dickinson, San Jose, CA). Aggregates were gated out and the percentage of cells in G2/M was calculated using Flow Jo software.

### Plasmid construction

The pCDNA3-myc.his vector (Invitrogen) was digested by Not1/Apa1 and different FANCJ fragments generated by PCR and digested by Not1/Apa1 were inserted. Primers are available upon request. Reverse primers used for K1249R-pCDNA3 and K1249RQ-pCDNA3 are 5′TTTTGGGCCCCCTAAAACCAGGAAACATGCC3′ and 5′TTTTGGGCCCCTGAAAACCAGGAAACATGCC3′, respectively. The K1249R and K1249Q pOZ vectors were generated with the QuickChange Site-Directed Mutagenesis Kit (Stratagene, La Jolla, CA) by using the FANCJ- pCDNA3-myc.his or FANCJ-pOZ as a template and the following primers: (K1249R-pOZ-Forward) 5′GGCATGTTTCCTGGTTTTAGGGCGGCCGCTGGAGGAGCA3′ and (K1249R-pOZ-Reverse) 5′GTCTCCTCCAGCGGCCGCCCTAAAACCAGGAAACATGCC3′; (K1239Q-pOZ-Forward) 5′GGCATGTTTCCTGGTTTTCAGGCGGCCGCTGGAGGAGAC3′ and (K1239Q-pOZ-Reverse) 5′GTCTCCTCCAGCGGCCGCCTGAAAACCAGGAAACATGCC3′; Recombinant FANCJ protein production was made in insect cells using the PVL13.2 vector as before [26]. Full-length WT *FANCJ* was used as a template to generate the acetylation mutants using the following primers: (K1249R-PVL132 Forward) 5′GGCATGTTTCCTGGTTTTAGGGACTACAAGGAGACG3′ and (K1249-PV132 Reverse) 5′CGTCGTCCTTGTAGTCCCTAAAACCAGGAAACATGCC3′. (K1249Q-PVL132 Forward) 5′ GGCATGTTTCCTGGTTTTCAGGACTACAAGGACGACG3′ and (K1249Q-PVL132 Reverse) 5′ CGTCGTCCTTGTAGTCCTGAAAACCAGGAAACATGCC3′. The pGEX-5X vector (GE Healthcare Life Sciences) was digested by Sal1/Not1 and the FANCJ C-terminal fragment was generated by PCR and digested by Sal1/Not1 and inserted. Primers are available upon request. All DNA constructs were confirmed by DNA sequencing.

### Viability assays

Stable FA-J cell lines were untransfected or transfected with siRNA previously described against Luc, Rad54, or polη [9]. Cells were seeded onto 6 well plates and incubated overnight. Seeded cells were either untreated or treated with increasing dose of MMC (1 h, serum free), UV, CPT, (1 h, serum free), or zeocin (1 h, serum free). To assay for percent survival, cells were counted 5–8 days post infection and percent survival was calculated as before [9].

### Helicase assays

Helicase assay reaction mixtures (20 µl) contained 40 mM Tris-HCl (pH 7.4), 25 mM KCl, 5 mM MgCl2, 2 mM dithiothreitol, 2% glycerol, 100 ng of bovine serum albumin/µl, 2 mM ATP, 10 fmol of 19-bp duplex DNA substrate (0.5 nM), and the concentrations of FANCJ (acetylated or non acetylated) indicated in the figures. Helicase reactions were initiated by the addition of FANCJ, and the reaction mixtures were incubated at 30°C for 15 min unless otherwise indicated. Reactions were quenched with the addition of 20 µl of 2× Stop buffer (17.5 mm EDTA, 0.3% SDS, 12.5% glycerol, 0.02% bromophenol blue, 0.02% xylene cyanol). For standard duplex DNA substrates, a 10-fold excess of unlabeled oligonucleotide with the same sequence as the labeled strand was included in the quench to prevent reannealing. Reaction products were resolved on nondenaturing 12% (19∶1 acrylamide-bisacrylamide) polyacrylamide gels, and quantitated as described previously [27].

### Immnuofluorescence microscopy

Stable FA-J cell lines were seeded onto 6 well plates and incubated overnight. Cells were either untreated or treated with 1 mM HU (24 h) or 0.25 µM CPT (1 h). Cells were fixed with 3% paraformaldehyde/2% sucrose for 10 min at RT, and permeabilized with 0.5% Triton X-100 in 20 mM HEPES for 5 min on ice. Incubation with antibodies and washes were described previously [6]. For Rad51 staining, cells were fixed with 3% paraformaldehyde/2% sucrose for 10 min at RT, permeabilized with ice-cold methanol for 30 min, and blocked with 4% BSA for 1 h. Staining was as described previously [6].

### *In vitro* acetylation assay

The acetyltransferase assays were performed in 30 µl of reaction, which includes reaction buffer (50 mM HEPES (ph 8.0), 10% glycerol, 1 mM DTT, 1 mM PMSF, 10 mM Na-butyrate), 1 µL [^3^H]-acetyl-CoA, 1 µl recombinant HAT domain of p300 (gift of Dr. Luo), and recombination FANCJ-CT or p53-CT [39]. Reaction were carried out at 30°C for 1 h and separated by SDS-PAGE, analyzed by autoradiography. Concentrations of recombinant proteins were determined by comassie staining from Invitrogen.

### In gel digestion

Gel bands containing FANCJ1 were de-stained twice with 25 mM ammonium bicarbonate in 50% acetonitrile for 30 min in 37°C, reduced with 7.6 mg/ml dithiothreitol at 60°C for 10 min, and alkylated with 18.6 mg/mL iodoacetamide at room temperature for 1 hour. The bands were then washed twice with 25 mM ammonium bicarbonate in 50% acetonitrile for 15 min at 37°C prior to shrinking with 50 µL acetonitrile for 10 min at room temperature. 100 ng trypsin (Promega) was added to each sample and 25 mM ammonium bicarbonate was added until the gels were fully swollen (∼10–50 µL) and the digestion proceeded overnight at 30°C. Following digestion, peptide extracts were transferred into new tubes and the gels were further extracted with 50 µL of 50% acetonitrile containing 5% formic acid (v/v) and following 15 min were added to the initial extracts. The latter process was repeated for a total of three extractions. Extracts were then dried on a SpeedVac and reconstituted in 20 µL of 0.1% formic acid for LC-MS/MS analysis.

### LC/MS/MS

Tryptic peptides (2 µL) were directly loaded at 4 µL/min for 7 min onto a custom-made trap column (100 µm I.D. fused silica with Kasil frit) containing 2 cm of 200 Å, 5 µm Magic C18AQ particles (Michrom Bioresources). Peptides were then eluted using a custom-made analytical column (75 µm I.D. fused silica) with gravity-pulled tip and packed with 25 cm 100 Å, 5 µm Magic C18AQ particles (Michrom). Peptides were eluted with a linear gradient from 100% solvent A (0.1% formic acid∶acetonitrile (95∶05)) to 35% solvent B (acetonitrile containing 0.1% formic acid) in 35 min at 300 nL/min using a Proxeon Easy nanoLC system directly coupled to a LTQ Orbitrap Velos mass spectrometer (Thermo Scientific) [40]. Data were acquired using a data-dependent acquisition routine of acquiring one mass spectrum from *m/z* 350–2000 in the Orbitrap (resolution 60,000) followed by tandem mass spectrometry scans in the LTQ linear ion trap of the 10 most abundant precursor ions found in the mass spectrum. Charge state rejection of singly-charged ions and dynamic exclusion was utilized to minimize data redundancy and maximize peptide identification [41].

### Data analysis

The raw data files were processed and searched against the human index of the SwissProt database (version 09/21/11) containing both the mutant and wild-type forms of FANCJ1 with Mascot (version 2.3.02; Matrix Science) using parent mass tolerances of 15 ppm and fragment mass tolerances of 0.5 Da. Full tryptic specificity with 2 missed cleavages was used and variable modifications of acetylation (protein N-term and lysine), pyro-glutamination (N-term glutamine), and oxidation (methionine), and fixed modification of carbamidomethylation (cysteine) were considered. Mascot search results were also loaded into Scaffold (Version 3.3.1; Proteome Software) for comparative analyses using spectral counting of tandem mass spectra and full annotation of the data [42].

## Supporting Information

Figure S1 FANCJ acetylation sites. Identified acetylation sites of FANCJ protein by mass spectrometry (bold): acetylated peptides unique to WT (red); unique to K1249R mutant (green); and common to both (black).
(EPS)

Figure S2 FA-J cells expressing FANCJ acetylation mutants have a distinct response to UV and zeocin induced damage. The FA-J cell lines were either untreated or treated with increasing doses of UV, zeocin, or CPT and allowed to grow for 8 days. Cells were counted and percent survival was calculated. Data represent mean percent ± s.d. of survival from three independent experiments.
(EPS)

Figure S3 FANCJ^WT^, FANCJ^K1249R^, and FANCJ^K1249Q^ have similar catalytic activities. FANCJ^WT^, FANCJ^K1249R^, and FANCJ^K1249Q^ recombinant proteins were incubated with forked duplex substrate at 30°C for 15 min. Reaction products were analyzed by nondenaturing gel electrophoresis. Data represent mean percent ± s.d. of growth from three independent experiments.
(EPS)

Figure S4 FANCJ is required for promoting CPT induced RPA phosphorylation. U2OS cells stably expressing shLuc or shFANCJ were either untreated or treated with CPT. Cell lysates were collected and analyzed with the indicated antibodies.
(EPS)

Figure S5 FANCJ is not required for promoting zeocin induced RPA phosphorylation. The FA-J cell lines were either untreated or treated with the denoted dose of zeocin. Cell lysates were collected and analyzed with the indicated antibodies.
(JPG)
