# Supplementary figures and images for: FANCJ/BACH1 Acetylation at Lysine 1249 Regulates the DNA Damage Response
Source: PLoS Genet. 2012 Jul 5;8(7):e1002786. doi: 10.1371/journal.pgen.1002786 (PMC3390368; doi:10.1371/journal.pgen.1002786)

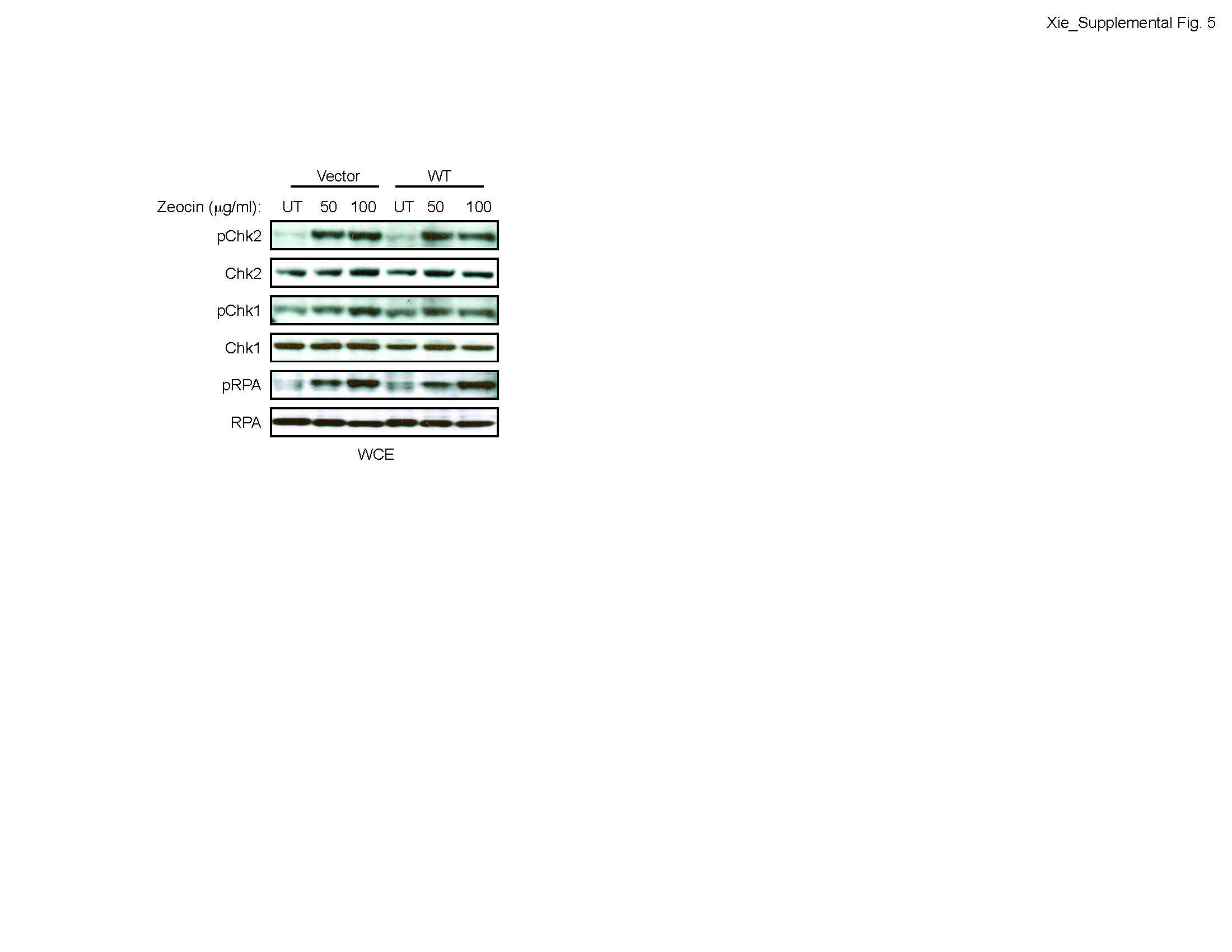

Supplement: Figure S5 — FANCJ is not required for promoting zeocin induced RPA phosphorylation. The FA-J cell lines were either untreated or treated with the denoted dose of zeocin. Cell lysates were collected and analyzed with the indicated antibodies. (JPG) [file pgen.1002786.s005.jpg]
